# Solid-state Stern–Gerlach spin splitter for magnetic field sensing, spintronics, and quantum computing

**DOI:** 10.3762/bjnano.9.147

**Published:** 2018-05-25

**Authors:** Kristofer Björnson, Annica M Black-Schaffer

**Affiliations:** 1 Department of Physics and Astronomy, Uppsala University, Box 516, S-751 20 Uppsala, Sweden; 2 Niels Bohr Institute, University of Copenhagen, Juliane Maries Vej 30, DK-2100 Copenhagen, Denmark

**Keywords:** Aharanov–Bohm, quantum computing, spintronics, Stern–Gerlach, SU(2), topological insulator

## Abstract

We show conceptually that the edge of a two-dimensional topological insulator can be used to construct a solid-state Stern–Gerlach spin splitter. By threading such a Stern–Gerlach apparatus with a magnetic flux, Aharanov–Bohm-like interference effects are introduced. Using ferromagnetic leads, the setup can be used to both measure magnetic flux and as a spintronics switch. With normal metallic leads a switchable spintronics NOT-gate can be implemented. Furthermore, we show that a sequence of such devices can be used to construct a single-qubit SU(2)-gate, one of the two gates required for a universal quantum computer. The field sensitivity, or switching field, *b*, is related to the characteristic size of the device, *r*, through *b* = *h*/(2π*qr*^2^), with *q* being the unit of electric charge.

## Introduction

Two famous examples of the fundamental difference between quantum mechanical and classical particles are provided through the Stern–Gerlach (SG) experiment [1] and the Aharanov–Bohm (AB) effect [2]. The SG experiment demonstrates the peculiar behavior of the quantum mechanical spin, teaching us that for any chosen axis the spin can be pointing either up or down. Even more nonintuitive, the spin can also be in a superposition of these two states, and thereby split in a SG apparatus to travel along different paths [1]. The AB effect, on the other hand, shows that the introduction of a magnetic vector potential has important effects on the phase of the wave function. This is not merely a mathematical formality, but has measurable consequences in interference measurements. When a particle travels along two different paths that enclose a magnetic flux, it picks up different phases along the two paths, even though the paths do not pass through the magnetic flux [2].

A topological insulator is a material with insulating bulk, but with topologically protected helical edge states. Here we show that it is possible to construct a solid state SG apparatus, or spin splitter, using the edge states in a two-dimensional topological insulator (2D TI) [3–13]. The device consists of a small hole drilled in the 2D TI, contacted by two leads. By threading a magnetic flux through the hole an AB-like effect gives rise to important interference effects, which allows for precise manipulation of spin currents, as has already been noted in [14]. While the ordinary AB effect arises because of interference in a single complex number, the effects achieved here relies on modifying the relative phase between the up and down components of the spin. Thus, the effects we describe here can be classified as a SU(2)-AB effects, while the ordinary situation corresponds to a U(1)-AB effect.

While the AB effect recently has attracted some attention in 3D TI [15–19], we here outline the concept for several concrete and different applications of the SU(2)-AB effect in a 2D TI. More specifically, we find that if using ferromagnetic leads, the device can be used for sensitive measurements of magnetic field strengths. The same setup can also be used to implement a spintronic switch. Instead using normal metallic leads, we show that a switchable spintronics NOT-gate can be constructed. Finally, we also demonstrate how a sequential setup of normal-lead solid-state SG spin splitters can be used to construct a single-qubit SU(2)-gate, one of two gates required to construct a universal quantum computer [20]. This also demonstrates the full extent to which the effect is best thought of as a generalization of the AB effect from U(1)-AB to SU(2)-AB.

## Results

### Setup

**Figure 1:**
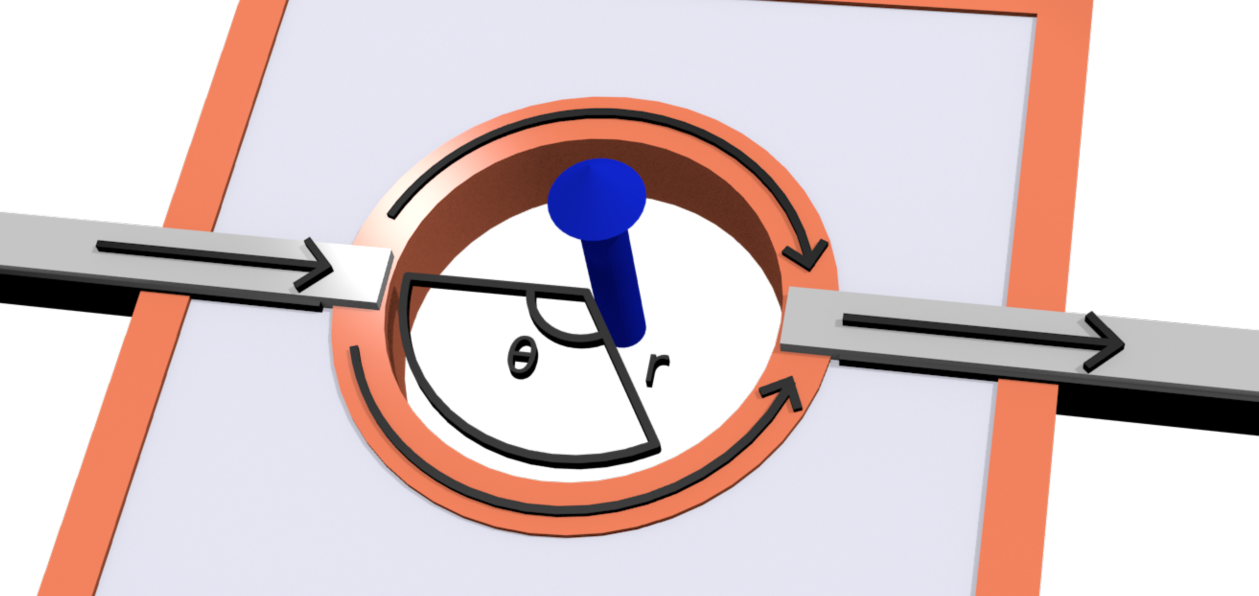
A hole drilled in a 2D TI creates two edge channels (orange). Leads (grey) are attached on each side of the hole, and a bias voltage is applied across the circuit. The transport properties of the device can be altered by threading a magnetic flux (blue arrow) through the hole, as well as by choosing either ferromagnetic or normal leads. The circular shape is not essential, but is used to simplify calculations.

Consider the conceptual setup in Figure 1. The circular channel around the hole forms an edge of the 2D TI and therefore hosts helical edge states. We assume for simplicity that the spin-polarization axis is perpendicular to the plane of the TI. The Hamiltonian describing the two counter-propagating edge channels is then simply given by


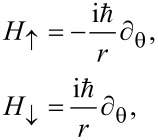


where arrows indicate the spin direction. In the ground state no net current is carried from one side to the other. Since the system is symmetric under a rotation of π around the *z*-axis orthogonal to the TI, even persistent currents are prevented. However, if a voltage is applied across the circuit, electrons can start to flow from one side to the other, say from the left to the right. This current will be proportional to the transfer matrix of the states that are occupied at the left side, but unoccupied on the right. We therefore begin by calculating this transfer matrix.

When considering processes that transfers electrons from the left to the right, we can, because of the helicity of the edge states, restrict ourselves to up-spins along the upper edge, and down-spins along the lower edge. Further, we introduce the coordinate *x*_1_ = *r*(2π − θ) and *x*_2_ = *r*θ along the upper and lower edges, respectively. The eigenvalue equations along the two edges are then


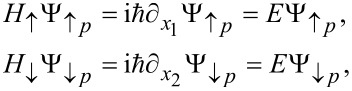


and the corresponding eigenstates can be written as


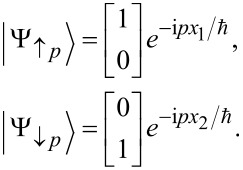


We now thread a magnetic flux of magnetic field strength *B* through the hole. To describe this we choose the vector potential 
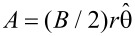
, which translates into 
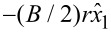
 and 
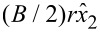
 in the new (*x*_1_, *x*_2_)-coordinates. The addition of this vector potential acts on the phase of the eigenstates according to


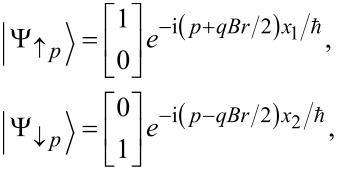


where *q* is the unit of electric charge. It is therefore clear that the transfer matrix that describes the transport of spins from the left side, *x*_1_ = *x*_2_ = 0, to the right side, *x*_1_ = *x*_2_ = *r*π, is given by

[1]
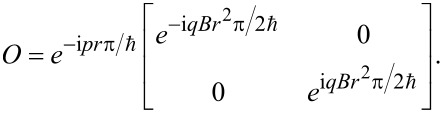


We here note that under a gauge transformation *A*→*A* + *A*′, where *A*′ satisfies 
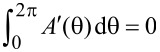
, the transfer matrix transforms as


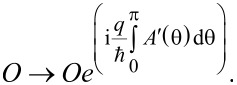


We have confirmed that this additional phase drops out of all physical quantities below, proving the gauge invariance of our results, and we can therefore set *A*′ = 0. Similarly, the overall phase in the above equation will drop out of all physical quantities. This also justifies us in not having specified the chemical potential. Because, as long as the spectrum is described by the same edge Hamiltonian, the only role of the chemical potential is to determine around which momentum *p_f_* the relevant excitations are located.

### Transfer between lead and edge channels

The total transfer matrix for the system will not only depend on the transfer matrix that describes the motion around the hole, but also on the matrices that describe the transfer processes between the leads and the circular edge. We will here assume that this process preserves phase coherence between the states in the leads and the TI edge states, and that it is described by a single tunneling parameter *t*, which we for now set to *t* = 1 to indicate perfect transmission between lead and edge. That is, the transmission is described by the identity matrix, and therefore contributes trivially to the total transfer matrix. However, we will in what follows be interested in tilting the TI by an angle φ relative to the quantization axis of the leads. It is therefore necessary to also let the total transfer matrix encode a change of basis between the leads and the TI. For this purpose we define two sets of coordinate axes, the laboratory axes *x*,*y*,*z*, and the TI axes *x*′,*y*′,*z*′. We choose to describe the electrons in the leads with the coordinates in the laboratory frame, while the edge states in the TI are described by the primed coordinates. It is clear that Equation 1 refers to the transfer of states in the primed basis. In particular, we choose the *x*,*x*′-axes along the direction of motion of the electrons through the circuit, while the *z*,*z*′-axes are chosen such that they coincide when φ = 0 and *z*′ is always perpendicular to the TI. Explicitly, the *x*,*y*,*z*- and *x*′,*y*′,*z*′-coordinates are related through


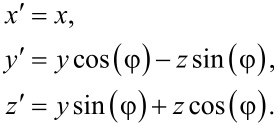


Using that spins transform according to


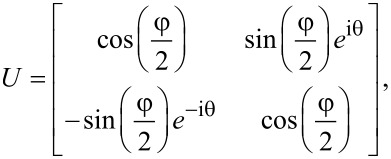


and simultaneously performing a gauge transformation *G* = diag(1, i) to simplify the expressions below, the change of basis from the *x*,*y*,*z*-basis to the *x*′,*y*′,*z*′-basis for the spins is given by

[2]
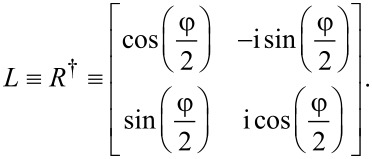


We have here used *L* and *R* to denote the transformations from the unprimed to the primed coordinates, and the primed to the unprimed coordinates, respectively. The symbols *L* and *R* are chosen since they are applied at the left and right end of the system, respectively. With these definitions we are now ready to write down the complete transfer matrix for the system


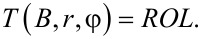


Here we have made explicit the dependence of *T* on the parameters *B* and *r* on Equation 1, and of φ on Equation 2. The main advantage of introducing the *L* and *R* matrices is that they allow us to work in the laboratory frame alone. To calculate the probability that an incoming spin σ in the left lead is transferred to a spin λ in the right lead, we now simply need to calculate the square of the corresponding matrix element


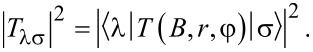


### Measuring magnetic flux

As a first example of a concrete application, we consider a system with fully spin-polarized ferromagnetic leads only containing electrons with spin-up. Further, the SG spin splitter is assumed to be oriented at an angle φ = π/2, which forces the incoming spins to split equally into both channels. Because the leads only conduct spin-up electrons, the only relevant matrix element for the scattering matrix is


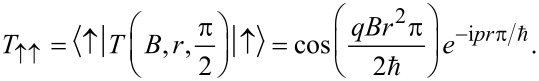


The conductance is therefore given by

[3]
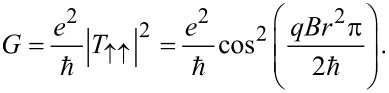


It is clear that the very strong dependence of the current on the magnetic flux *Br*^2^π makes this setup ideal for measuring magnetic field strength, as a potential alternative to superconducting quantum interference devices (SQUIDs). The measurement resolution is directly set by the radius of the hole in the TI. This is of special interest because it provides a potential route for high-resolution magnetic field measurements even at room temperatures [21–22].

### Logic spintronics gates

Next we note that the configuration in the previous section can also be used as a spintronics switch, with voltage used to encode 0 and 1. The two leads can be used as source and drain, while the magnetic field is used as the gate. From Equation 3 it is clear that a magnetic field strength 
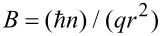
 corresponds to “on” and “off” states for *n* even and odd, respectively, and we therefore define the magnetic switching quantum

[4]
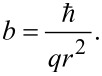


An alternative way to encode 1 and 0 is to use the currents of up- and down-spins, respectively. This requires normal leads through which both up- and down-spins can be transported. We therefore consider the same configuration, but now evaluate all four components of the transfer matrix *T*(*B*, *r*, π/2):


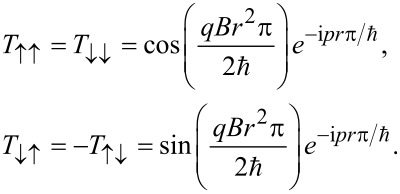


Similarly to the expressions above, the square of the transfer matrices gives the transfer probability of the spin-polarized currents. In particular, the off-diagonal matrix elements 
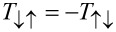
 converts between up and down spin currents. Therefore, the device relates the ingoing and outgoing spin currents to each other through


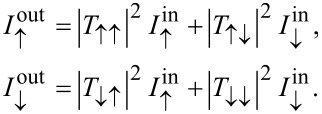


Considering once again the special case 
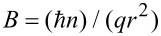
, with *n* being an integer, the currents transforms according to


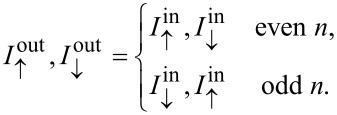


This means that the device can be switched between a normal lead and a NOT-gate, simply by changing *B* by the switching quantum in Equation 4.

### Quantum computer gate

Having seen how a TI SG apparatus can be used to construct classical logic gates for spintronics, we finally turn to possible applications in quantum computing. It has been shown that a universal quantum computer can be built using only two-qubit CNOT-gates and single-qubit SU(2)-gates [20]. We here show that a SG TI spin-splitter provides a route for implementing the latter of these two gates.

**Figure 2:**
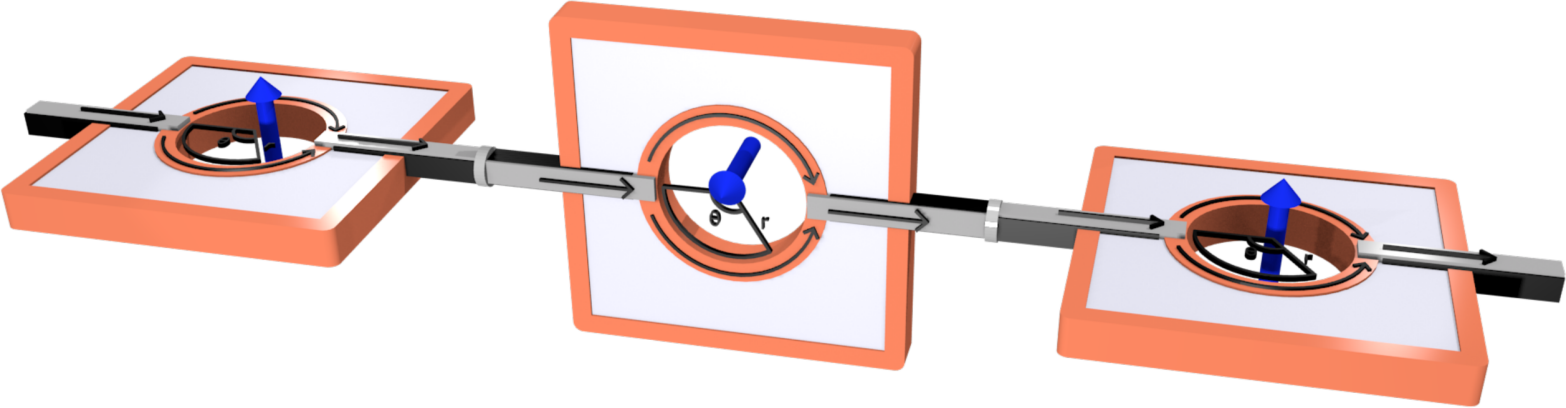
Three solid-state SG spin splitters in series, with the middle device at an angle π/2 relative to the other two.

For this purpose we consider three sequential spin-splitters connected by normal leads. The three devices are oriented as in Figure 2, with the middle device oriented at an angle φ_2_ = π/2, while the first and the last spin splitter are at an angle φ_1_ = φ_3_ = 0. The total transfer matrix for the complete system is then given by


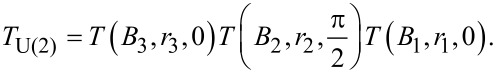


When evaluated, this expression can be written as

[5]
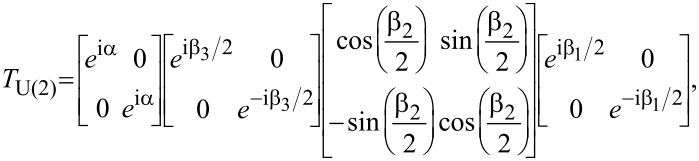


where


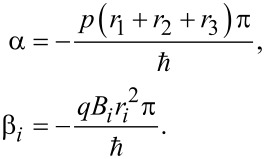


The six physical parameters *B_i_*, *r_i_* are more than sufficient to make the four parameters α, β_1_, β_2_ and β_3_ independent of each other. Moreover, when all these four parameters can be chosen independently, it is possible to express any U(2)-matrix using Equation 5 [20]. Thus, it is possible to implement any unitary single-qubit gate, and in particular any SU(2)-gate, through the use of three sequential solid-state SG spin splitters. In fact, the overall U(1)-phase provided by the parameter α can be ignored for reasons similar to those for which the U(1)-phase provided by the gauge transformation *A*→*A* + *A*′ can be ignored. This phase would only be relevant if the incoming electron is further split up into one part passing through the device, and one part moving through another path joining only at the far right outgoing lead.

In light of these results it is useful to think of the devices discussed here as exhibiting an SU(2)-AB effect. While the ordinary AB effect arises as a consequence of interference in a single U(1)-phase, these devices rely on a generalized SU(2)-interference effect in the relative phase and amplitude of the up- and down-components of the spin. To be able to create an arbitrary SU(2)-transformation, a sequence of three devices is needed, while an individual spin splitter gives rise to a subset of such SU(2)-transformations. Finally, we note that in this calculation we have omitted transfer matrices describing the propagation through the leads. We are justified in doing so because these would be proportional to the identity matrix and therefore only contribute to the irrelevant α phase.

## Discussion

We would like to end with a few comments on some of the assumptions made when deriving the above results. First of all, the tunneling parameter *t*, which otherwise would have multiplied the *L* and *R* matrices was set to *t* = 1. It is clear that the zero-th order correction to deviations from *t* = 1 is to include the factor *t*^2^ in front of all transmission coefficients, which shows up as *t*^4^ in the conductivity. The higher-order corrections would come from particles that are reflected and travel an additional time around the loop. While such terms can introduce corrections to the interference pattern for intermediate field strengths, they would not affect the result at multiples of the switching quantum in Equation 4. The reason for this is that additional circuits around the loop will only affect the relative phase between the up- and down-spins by multiples of 2π. Such interference effect could also play a role for *t* = 1 when ferromagnetic leads are used, because the down spins at the right edge will be completely reflected. In a standard Landauer treatment such reflected terms would have been taken into account through reflection matrices in addition to the transmission matrix we have derived, as was for example done in [14]. However, a 2D TI is very special in this regard, because the reflected spins travel back along the opposite edge from which it traveled toward the exit lead. Since we are only interested in forward propagation of up spins along one edge, and down spins along the other, it is possible to add additional floating ferromagnetic leads with opposite spin polarization to the forward propagating modes to the two edges. This allows for reflected spins to escape without affecting the forward propagating spins and thereby we can suppress higher-order corrections.

We also mention that although the setup in Figure 2 might seem difficult to realize in practice, the focus of this work is to provide a conceptual setup and an explanation of the phenomenon itself. In fact, the only reason the middle spin splitter is tilted at an angle π/2 is to make its edge states have their spin-polarization perpendicular to those of the other two. In practice it would therefore be possible to have all three devices in the same plane, if it is constructed out of two different types of 2D TIs with perpendicular spin-polarization axes.

## Conclusion

We have shown that the helical edge states of a 2D TI can be utilized to construct a solid-state SG spin splitter that when threaded by a magnetic flux gives rise to a generalized SU(2)-AB interference effect. With two ferromagnetic leads, the device can be used to accurately measure magnetic flux, as well as be used as a magnetic field gated spintronics switch. Instead by using normal leads, a switchable spintronics NOT-gate can be implemented, or when using three devices connected in sequence, a SU(2)-gate for quantum computing is achieved.
